# Biodegradable Redox-Sensitive Star Polymer Nanomicelles for Enhancing Doxorubicin Delivery

**DOI:** 10.3390/nano9040547

**Published:** 2019-04-04

**Authors:** Meng Li, Jian-Wei Guo, Wei-Qiu Wen, Jem-Kun Chen

**Affiliations:** 1 School of Chemical Engineering & Light Industry, Guangdong University of Technology, Guangzhou 510006, China; limenglcm@163.com (M.L.); Will_Wenwq@163.com (W.-Q.W.); 2 Department of Materials Science and Engineering, National Taiwan University of Science and Technology, No. 43, Sec. 4, Keelung Road, Taipei 106, Taiwan

**Keywords:** adamantane, star, redox-sensitive, nanomicelles, biodegradable polymer

## Abstract

A typical amphiphilic star polymer adamantane-[poly(lactic-co-glycolic acid)-bis(2-carboxyethyl) sulfide-poly(ethylene glycol) monomethyl ether)]_4_ with a specific hydrophilic/redox-sensitive/hydrophobic structure was designed and synthesized through ring opening and esterification reactions. The self-assembled nanomicelles were used as doxorubicin (DOX) delivery vehicles with suitable critical micelle concentrations (5.0 mg/L). After the drug being loaded, drug-loaded micelles showed good drug-loading efficiency (10.39%), encapsulation efficiency (58.1%), and drug release (up to 60%) under simulated biological environment conditions. In addition, the backbone structure of the biodegradable polymer was easily hydrolyzed by the action of biological enzymes. As expected, cell-based studies showed that the designed polymer micelles possessed good biocompatibility (a survival rate of 85% for NH-3T3 cells). Moreover, the drug (DOX) still maintained good anti-cancer effects after being loaded, which caused 40% of MCF-7 cells to survive. These redox-sensitive micelles showed anti-tumor therapeutic potential.

## 1. Introduction

In recent years, polymer micelles in nano drug delivery systems have attracted much attention in the chemotherapy of cancer [1,2]. Polymers with highly branched, complex topologies (such as dendritic, hyperbranched, multi-arm star-shaped, and brush copolymers) usually have unique physicochemical properties that have been applied in the fields of biology and materials [3,4]. Multi-arm star polymers have higher solubility, lower viscosity, and superior thermodynamic properties compared with linear polymers, which have an easier synthesis process and milder reaction conditions than dendritic and hyperbranched copolymers. In addition, star-shaped polymers formed by self-assembly with core-shell micelles can encapsulate drugs in the core and effectively protect the system over long periods of time to reduce reticuloendothelial system (RES) clearance and renal filtration [5]. During the circulation process in the body, drug-loaded micelles can be accumulated at the tumor cells site because of different pressure and retention effects [6,7,8], while the release of the junction is triggered by biostimulation (e.g., pH, redox or light) [9,10,11,12]. Glutathione (GSH) is one of the most abundant tripeptide molecules containing thiol groups in cells, which can be involved in the cleavage of disulfide bonds in organisms. Since the concentration of GSH is significantly different between cancer cells (10 mM) and normal tissue (2 µM), polymer micelles with disulfide bonds are commonly used to control the release of anticancer drugs, as they allow the drug to be released under certain conditions [13,14,15].

It is known that adamantane is an existing non-toxic and tasteless chemical [16]. The introduction of an adamantane skeleton can enhance the fat-soluble effect of its derivative, which allows the new polymer to encapsulate more hydrophobic drugs during the formation of self-assembled micelles because of the interaction between hydrophobic molecules. Moreover, adamantane has become a good choice in clinical practice because of the rigidity and stability of its structure. In our previous work [17], the highly stable adamantane-based star polymer adamantane-[poly(ε-caprolactone)-*b*-poly(2-(diethylamino)ethyl methacrylate)-*b*-poly(poly(ethylene glycol) methyl ether methacrylate)]_4_ (Ad-(PCL-b-PDEAEMA-b-PPEGMA)_4_) micelles were developed for controlled drug delivery, and the drug-loaded micelles release 67% of doxorubicin (DOX) under acidic conditions. Therefore, it is feasible to introduce adamantane as a core into an amphiphilic polymer.

**Scheme 1:**
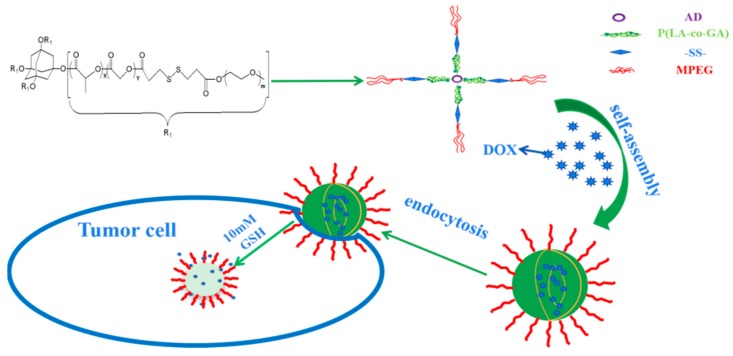
Encapsulation and release of doxorubicin (DOX) by polymer adamantane-[poly(lactic-co-glycolic acid)-bis(2-carboxyethyl) sulfide-poly(ethylene glycol) monomethyl ether)]_4_ micelles.

Biodegradable block copolymer is a promising drug-carrier material, with physical and chemical properties that can be regulated by adjusting the block composition ratio or adding a new block that meets the requirements [11,18,19]. Degradable materials (polycaprolactone (PCL), polyglycolide (PGA), polylactide (PLA), poly(lactic-co-glycolic acid) (PLGA), etc.) have been widely used as sustained release carriers with long-lasting effects due to their good biocompatibility and biodegradability [20]. In our work, two different star-shaped triblock polymers (DL-lactide (DL-LA):glycolide (GA) = 60:20 or 75:25) were designed to maintain degradation efficiency. In order to improve the stability of the micelles, adamantane and PLGA were introduced into the hydrophobic section. The introduction of a disulfide bond into the polymer allowed the encapsulated drug to be targeted for release. Compared with pentaerythritol as the core polymer [21,22], a redox-sensitive polymer with better performance, namely adamantane-[poly(lactic-co-glycolic acid)-bis(2-carboxyethyl) sulfide-poly(ethylene glycol) monomethyl ether)]_4_ (AD-[P(LA-co-GA)-SS-mPEG]_4_), was designed as a drug delivery material for DOX delivery and release (Scheme 1).

## 2. Materials and Methods

### 2.1. Materials

1,3,5,7-Tetrahydroxyadamantane (AD-(OH)_4_) was prepared according to the reported literature [23]. DL-LA, GA, pentaerythritol (PT), stannous octoate (Sn(Oct)_2_), pyrene, N,N-dimethylformamide (DMF, 99.9%, extra dry, water ≤ 30 ppm), 3,3′-dithiodipropionic acid, and triethylamine were purchased from Energy Chemical (Shanghai, China). DL-dithiothreitol (DTT), 4-dimethylaminopyridine (DMAP), poly(ethylene glycol) monomethyl ether (mPEG, M_W_ = 2000 g/moL), doxorubicin hydrochloride (DOX·HCl), and dicyclohexylcarbodiimide (DCC) were obtained from Aladdin Bio-Chem Technology (Shanghai, China). Dimethyl sulfoxide (DMSO), dichloromethane, petroleum ether, deionized water, and ethanol were purchased from Damao Chemical (Tianjin, China). All chemicals can be used directly without further processing or purification.

### 2.2. Synthesis of Amphiphilic Polymers

**Scheme 2:**
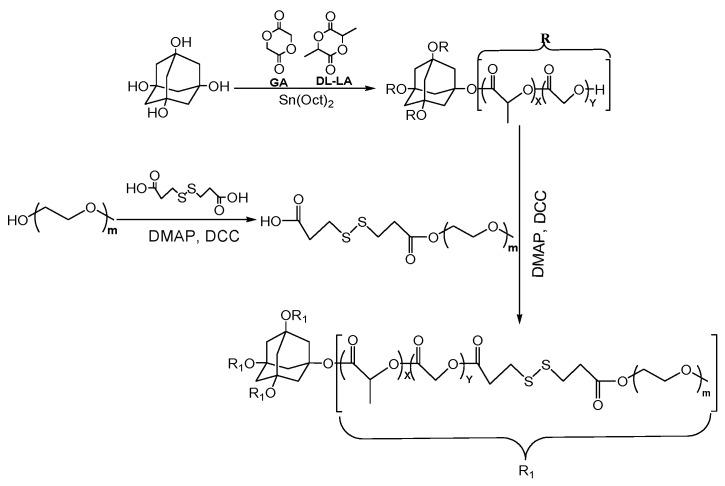
The synthetic route of the AD-[P(LA-co-GA)-SS-mPEG]_4_.

The amphiphilic polymers including AD-[P(LA_60_-co-GA_20_)-SS-mPEG]_4_, PT-[P(LA_75_-co-GA_25_)-SS-mPEG]_4_, and AD-[P(LA_75_-co-GA_25_)-SS-mPEG]_4_ were synthesized via ring-opening polymerization (ROP) and continuous esterification reactions [24,25,26,27], and named as polymer 1, polymer 2, and polymer 3, respectively. The synthetic characterization and performance analysis of polymer 3 are shown below as an example, and the synthetic route of polymer 3 is presented in Scheme 2.

### 2.3. Synthesis of AD-P(LA-co-GA)_4_ Copolymer

AD-P(LA-co-GA)_4_ block copolymer was synthesized via ring-opening polymerization of DL-LA and GA in the presence of AD-(OH)_4_ and Sn(Oct)_2_ as initiator and catalyst, respectively. Briefly, AD-(OH)_4_ (0.08 g, 0.4 mmol) was firstly dried in a Schlenk flask (Wattecs, Xian, China) under vacuum at 100 °C for 8 h to remove the residual moisture. Then, DL-LA (4.32 g, 30 mmol), GA (1.16 g, 10 mmol), and Sn(Oct)_2_ (40 µL, 0.12 mmol) were added into the flask. Subsequently, the flask was evacuated and flushed with argon (Special Gas, Guangzhou, China) three times, and immersed into an oil bath at 130 °C for 12 h. The crude products were dissolved in 30 mL dichloromethane, and added dropwise to cold petroleum ether to precipitate the product. The precipitate was washed with cold petroleum ether and ethanol three times, respectively, and dried under vacuum for 48 h (yield: 65%).

### 2.4. Synthesis of MPEG-SS-COOH and AD-[P(LA-co-GA)-SS-mPEG]_4_

3′3-Dithiodipropionic acid (2 molar equivs), DCC (4 molar equivs), and DMAP (0.4 molar equivs) were dissolved in 25 mL anhydrous DMF. The mixture was evacuated and flushed with argon three times and stirred for 2 h in an ice bath to activate the carboxyl of the 3,3′-dithiodipropionic acid. After adding mPEG (1 molar equiv), the reaction was continued at room temperature for another 48 h. Then, the mixture was transferred to a dialysis membrane (molecular weight cut off (MWCO) 1500 Da) and dialyzed against deionized water for 48 h to remove unreacted compounds, changing the deionized water every 6 h. After dialysis, the supernatant fluid in the dialysis membrane was collected and further freeze-dried for 48 h to obtain the product mPEG-SS-COOH (yield: 52%). Similarly, the final product AD-[P(LA-co-GA)-SS-mPEG]_4_ was obtained with the same synthetic esterification method (yield: 40%).

### 2.5. Characterization

The ^1^H NMR spectra of the polymers was obtained with a Bruker AVANCE III 400 MHz superconducting Fourier (Bruker, Billerica, MA, USA) at 25 °C, and 10 mg per milliliter polymer in deuterated chloroform (CDCl_3_, Meryer Technologies, shanghai, China ) were measured. The number average molecular weight (M_n_) and distribution coefficients (M_w_/M_n_) of the polymers were analyzed by gel permeation chromatography (GPC) with a Waters 1525 system (Waters, Milford, MA, USA) equipped with a liquid chromatograph (LC) quant pump and refractive index (RI) detector. Tetrahydrofuran was used as eluent (1 mL/min), and monodisperse polystyrene (20 μL) was used as the standard.

### 2.6. Preparation of Self-Assembled Amphiphilic Polymer Micelles

The polymer micelles self-assembled in an aqueous solution were prepared using a dialysis method. A total of 10 mg polymer dissolved in 5 mL DMSO was firstly transferred to a dialysis membrane (MWCO 10000 Da) and dialyzed against deionized water for 48 h. Then, 10 mL solution was prepared in a 10-mL volumetric flask. Finally, the polymer micelles solution (1 mg/mL) was obtained using a 0.45-µm syringe filter with polyethersulfone (Keyilong, Tianjin, China) and stored in a refrigerator at 4 °C.

### 2.7. Critical Micelle Concentration (CMC) Measurement

The CMC value of the polymer micelles was determined by a FluoroMax-4 fluorescence spectrometer (HORIBA Jobin Yvon, Clifton Park, NY, USA) using pyrene as a fluorescent probe. The fluorescence scan range was from 300 to 350 nm and the emission wavelength was 334 nm. The polymer was dissolved in deionized water and then diluted to a range of concentrations from 0.0001 to 0.1 mg/mL with deionized water. Then, a pyrene solution with a concentration of 5.94 × 10^−7^ M was added to 5 mL polymer micelles solution, and the mixture was equilibrated in the dark at room temperature for 24 h.

### 2.8. Determination of Particle Size and Polydispersity (PDI) of Polymer Micelles

A certain amount of polymer micelles solution (1 mg/mL) was added to the sample cuvette, and particle sizes and PDI of the micelles were measured by a BI-200SM dynamic light scattering (DLS) particle size analyzer (Brookhaven, New York, NY, USA). Meanwhile, in order to investigate the redox response behavior of polymer micelles at different DTT concentrations, polymer micelles and drug-loaded micelles were incubated in a phosphate-buffered saline (PBS) containing 0 mM and 10 mM DTT at 37 °C, respectively. The mixture was taken out after 8 h, and the change in the particle sizes of the mixture was measured by DLS.

### 2.9. Preparation of DOX-Loaded Micelles and Observation of Morphology

A certain amount of DOX·HCl and triethylamine (1.5 molar equivs) were dissolved in 10 mL DMSO and stirred overnight at a speed of 300 rpm to allow HCl to be reacted completely. After adding polymer and stirring at room temperature for 2 h, the solution was transferred to a dialysis membrane (MWCO 10000 Da) and dialyzed against deionized water for 48 h to remove free DOX. The supernatant liquid was freeze-dried to obtain a solid powder. The solutions of micelles were separately dropped onto a 300-mesh ordinary carbon support film. After the solvent naturally volatilized, the morphology and structure of the micelles were observed using a TalosF200S transmission electron microscopy (TEM, FEI, Hillsboro, OR, USA).

### 2.10. In Vitro Release Experiment of Drug-Loaded Micelles

Three portions of 10 mL DOX-loaded micelles (1 mg/mL) were separately transferred to three dialysis membranes (MWCO 10000 Da) and incubated in 40 mL of PBS (pH = 7.4) containing different concentrations of DTT, which were 0 mM, 5 mM and 10 mM, respectively. The redox-dependent DOX release measurement was carried out at a speed of 180 rpm in a water bath at a constant temperature of 37 °C. At a predetermined time (0, 2, 4, 6, 8, 16, 32, and 48 h), 2 mL of the incubated solution was taken out, and 2 mL of fresh PBS containing the corresponding DTT concentration was added to refill the culture to 40 mL. The redox-dependent DOX release profile was determined from the absorbance of the solution in an ultraviolet and visible spectrophotometer (UV-2450, Shimadzu, Japan) at 483 nm. All DOX release experiments were tested in parallel three times, and the cumulative release value (%) of DOX was calculated using Formula (1). To determine the drug-loading efficiency (DLE) and drug-encapsulation efficiency (DEE) using Formulas (2) and (3), the freeze-dried DOX-loaded micelles powder was dissolved in PBS and analyzed by the UV-2450 spectrophotometer. Calibration curves were obtained using DOX/PBS solutions with different DOX concentrations and intensity.

(1) $$E_{r}=\;\frac{V_{e}\sum_{1}^{n-1}c_{i}\;+\;c_{0}V_{0}}{m_{DOX}}\;\times \;100\%$$

where *V_e_* = 10 mL; *V*_0_ = 40 mL; *c_i_* was the sample concentration at the *i*-th replacement sample; *m_DOX_* represented the amount of DOX in the micelles.

(2) DLE(wt.%)=(wt of loaded drug/wt of polymer) × 100%

(3) DEE(wt.%)=(wt of loaded drug/wt of feeding drug)×100%

### 2.11. Cytotoxicity Test

The toxicity of polymer micelles and DOX-loaded micelles was evaluated by measuring the survival rate of mouse embryonic fibroblasts (NIH-3T3) cells and human breast cancer (MCF-7) cells by Cell Counting Kit-8 (CCK-8) assay. NIH-3T3 and MCF-7 cells were seeded in 96-well plates in 80 µL medium at a density of 5000 cells/well, and incubated for 12 h at 37 °C in an equilibrium atmosphere of 5% CO_2_. The same amount of fresh medium containing different concentrations of free DOX, blank micelles, and DOX-loaded micelles were used to replace the used medium, and the cells were cultured for an additional 48 h. In the CCK-8 assay of NIH-3T3 cells, the concentration range of polymer micelles and free DOX was 1.23–300 µg/mL. The concentration range of DOX-loaded micelles and free DOX was 0.032–100 µg/mL in the CCK-8 assay of MCF-7 cells. Relative cell viability was measured by CCK-8 assay and the absorbance of the solution was measured at 450 nm on a VICTOR Nivo multimode plate reader (PerkinElmer, Shanghai, China).

## 3. Results and Discussion

### 3.1. Characterization of Polymer 3

**Figure 1:**
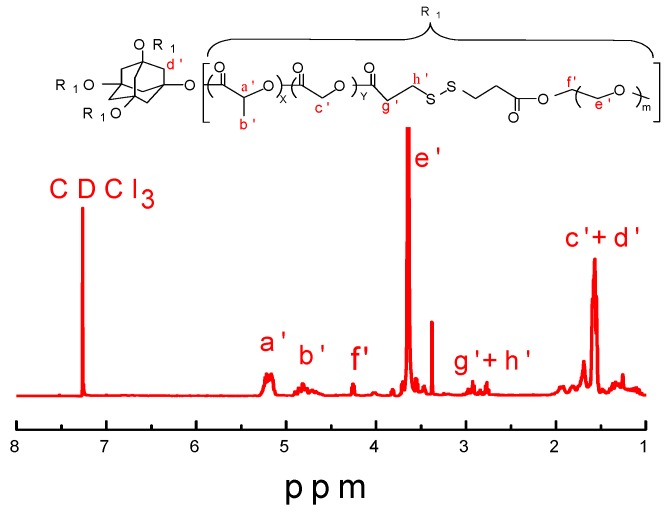
^1^H NMR spectrum of polymer 3 in CDCl_3_.

**Figure 2:**
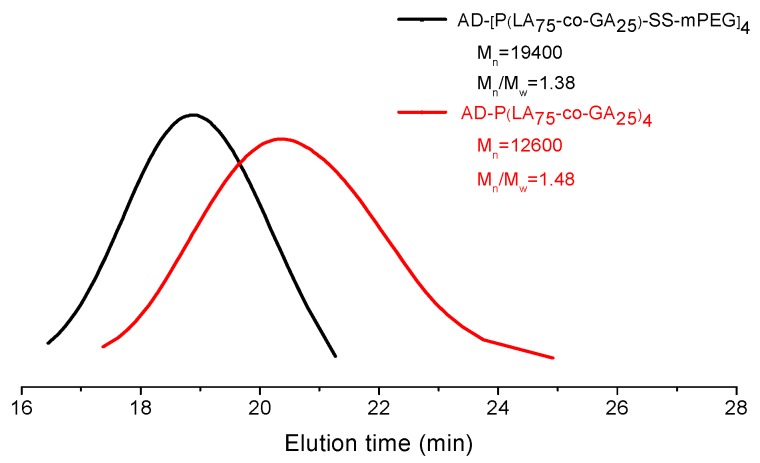
GPC of polymer 3 and AD-P(LA_75_-co-GA_25_)_4_.

The structure of polymer 3 was confirmed by ^1^H NMR spectrum shown in Figure 1. The strong signal peak indicated the proton of the methylene group of the repeating unit of mPEG, and its absorption peak position was 3.67 ppm. The proton signal of P(LA-co-GA) appeared at 1.55–1.65 ppm, 4.7–4.9 ppm, and 5.1–5.3 ppm. In addition, the peaks at 2.75 ppm and 2.92 ppm belonged to -CH_2_CH_2_-S-S-CH_2_CH_2_- groups, indicating that the hydrophilic and hydrophobic segments were successfully linked. Therefore, it was confirmed that polymer 3 was successfully synthesized. The M_n_ and M_w_/M_n_ of the three polymers were characterized by GPC analysis (Table 1), and the M_n_ of each polymer was similar to the molecular weight expected and calculated by ^1^H NMR spectroscopy. We found that the M_n_ values of the synthesized polymers 1, 2, and 3 increased by 6500–7500 compared with their precursors, indicating that the esterification reaction on the four arms of the polymer molecule was relatively average in general. Therefore, it can be analyzed from the molecular weight of the polymer in GPC that the structure of the polymer was star-shaped, which is consistent with our expected molecular composition and structure. Figure 2 indicated that polymer 3 and AD-P(LA-co-GA)_4_ had narrow molecular weight distributions (M_w_/M_n_ < 1.5), because the synthetic process was controllable.

### 3.2. Self-Assembly of Amphiphilic Polymer 3

**Figure 3:**
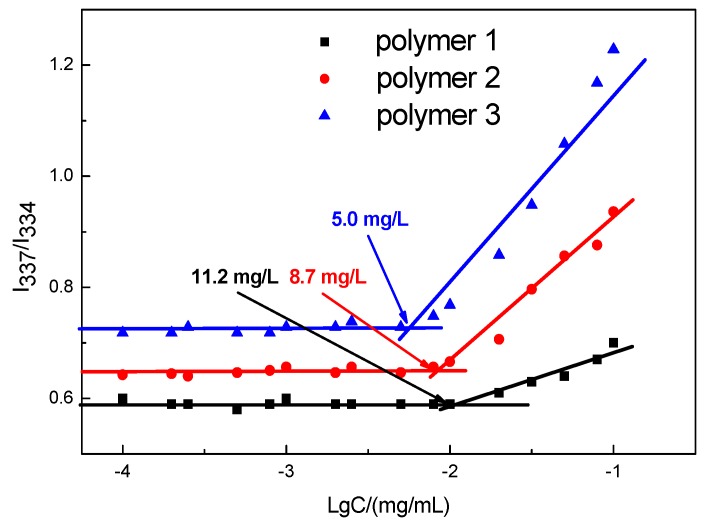
Self-assembled critical micelle concentration (CMC) of polymers 1, 2, and 3.

The synthesized amphiphilic polymer micelles can be spontaneously formed in aqueous solution driven by the strong hydrophobic/hydrophilic interaction between the linear chains of the AD-(PLGA)_4_ core and mPEG shell [28,29]. The pyrene was used as a probe to determine the CMC value of the polymer by fluorescence spectrometer. In general, the CMC value can reflect the self-assembly ability of the polymer to form micelles, and a lower CMC value is desired for increasing micellar stability in the blood stream [30,31]. From Figure 3, the CMC values of the three polymers were determined from the crossover points ranging from 5.0 to 11.2 mg/L, consistent with the high stability of polymer micelles; the CMC values of polymer 1, polymer 2 and polymer 3 were 11.2 mg/L, 8.7 mg/L, and 5.0 mg/L, respectively. This phenomenon can be attributed to the hydrophobic interaction of the polymer and the length of the hydrophobic segments [32,33]. Compared with polymer 2, polymer 3 had a lower CMC value, which is mainly due to the introduction of the adamantane unit with a superior lipophilicity structure.

**Figure 4:**
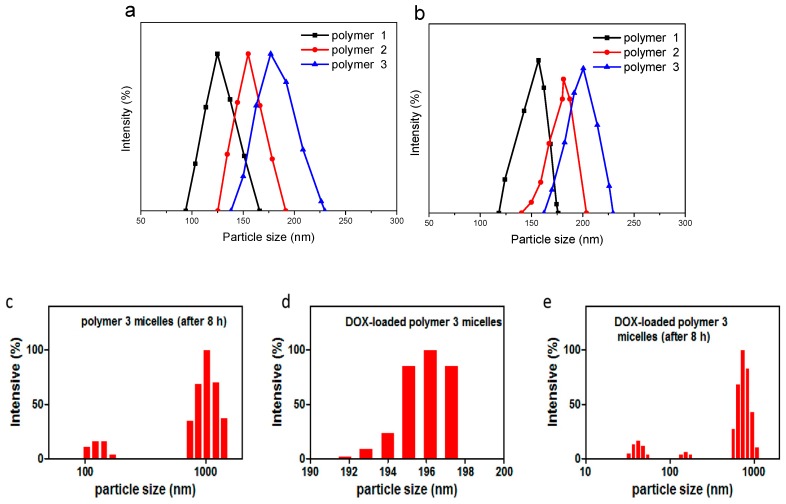
Particle size distributions of micelles of the three polymers in PBS for (**a**) 0 h and (**b**) 8 h; (**c**) polymer 3 micelles’ response to 10 mM DTT in PBS for 8 h, (**d**) Dox-loaded polymer 3 micelles; and (**e**) drug-loaded polymer 3 micelles’ response to 10 mM DTT in PBS for 8 h.

The redox sensitivity of the synthesized polymer was confirmed by DLS tests (Figure 4). The average particle sizes of three polymer micelles are shown in Figure 4a, and were about 120 nm, 156 nm, and 175 nm, respectively. Due to the longer hydrophobic segment P(LA-co-GA), the particle sizes of polymer 3 were larger than those of polymer 1, which had a larger hydrophobic core. Compared with polymer 2, the core of the three-dimensional structure (adamantane) and the interaction between hydrophobic adamantane and PLGA hydrophobic segments might be the reason for the larger size of polymer 3 micelles. In addition, in order to study the stability of the polymer micelles under normal physiological conditions, polymer 3 micelles were allowed to stand in PBS at 37 °C for 8 h. Figure 4b shows that these micelles still exhibited excellent stability. The reason why the micelle particle size was slightly larger may be the instability of the ester group of the polymer in the PBS solution for 8 h, but the protection of the hydrophilic shell kept the micelle in a relatively stable state. To investigate the in vitro stability of polymer 3 micelles and DOX-loaded polymer 3 micelles under reducing conditions, they were separately incubated in PBS containing 10 mM DTT with continuous oscillation for 8 h. In the presence of 10 mM DTT (Figure 4c,e), the particle size distribution after 8 h was more uneven than the initial distribution (Figure 4a,d). Because the cracked disulfide bond of the polymer caused the hydrophilic shell to be broken and the hydrophobic fragments to aggregate in the PBS containing 10 mM DTT, when the micelles structure and the disulfide bond were destroyed, the heterochain polymer with many ester bonds could be hydrolyzed in the aqueous solution due to the polarity of the main chain, which was also the reason for the emergence of small molecules [34].

**Figure 5:**
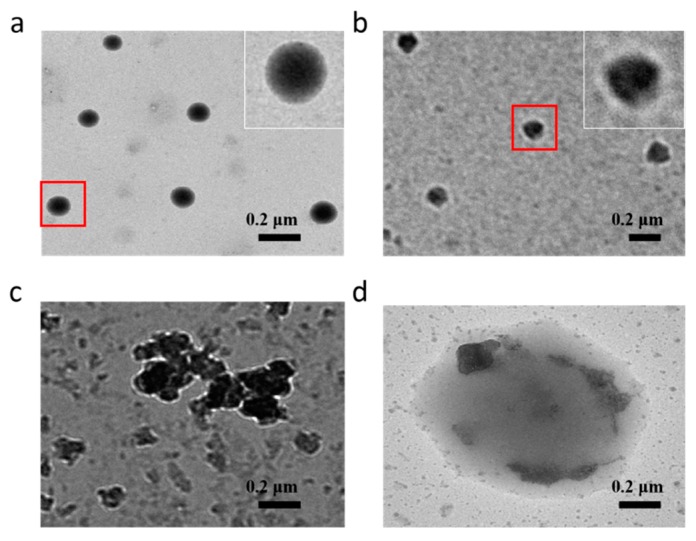
Typical TEM images of (**a**) polymer 3 micelles, (**b**) DOX-loaded polymer 3 micelles, (**c**) polymer 3 micelles in PBS (10 mM DTT), and (**d**) DOX-loaded polymer 3 micelles in PBS (10 mM DTT).

The TEM images of polymer 3 micelles and drug-loaded polymer 3 micelles, as presented in Figure 5a,b, showed two core-shell spheres with the diameters of less than 170 nm. The average particle size of the micelles increased slightly after the drug was loaded, as the drug was embedded in the core of the micelles and adsorbed on their surface. However, the diameters of polymer 3 micelles and drug-loaded polymer 3 micelles in the TEM image were smaller than those in DLS tests because of the volatilization of the solvent during the sample preparation for TEM. The redox response behavior of the micelles can also be studied by TEM. Figure 5c shows a TEM image of polymer micelles incubated in PBS solution containing 10 mM DTT, where the micelles with irregular shape gathered. This can be explained the fact that the hydrophilic shells of the micelles start being destroyed, and the hydrophobic groups gathered and resulted in aggregation of the micelles. The DOX released from the irregularly shaped micelles can be observed in Figure 5d, which indicates that the aggregated drug micelles had decomposed. At a high concentration of DTT, the cleavage of disulfide bonds caused drug release and the aggregation of hydrophobic macromolecules. More importantly, the process of drug release was concurrent with the process of aggregation, which is consistent with the results of the DLS test and the in vitro release test.

### 3.3. In Vitro Release of DOX from Micelles

It can be seen from Table 2 that the DLE and DEE of DOX-loaded polymer 3 micelles were 10.39% and 58.1%, respectively. The average size of DOX-loaded polymer 3 micelles was 196 nm in DLS tests, and it increased by more than 22 nm compared to polymer 3 micelles. This may be caused by the interaction between hydrophobic DOX and hydrophobic segments. Furthermore, encapsulated DOX also led to larger particle sizes. For the three polymers, the results indicate that the longer hydrophobic segment can enhance the ability to carry the drug. Generally, more hydrophobic fragments will form larger cores in micelles to encapsulate more drug. The drug-loaded micelles with adamantane as the core had greater drug loading and encapsulation efficiencies under the same conditions, which might be attributed to the three-dimensional structure of the core and excellent lipophilicity of adamantine. Therefore, the polymer 3 micelles with better performance were selected for drug release experiments and in vitro cell tests.

**Figure 6:**
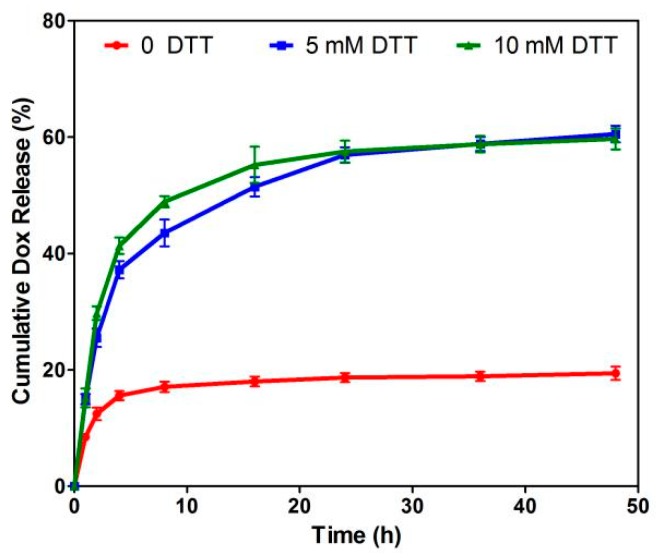
In vitro drug release of DOX-loaded polymer 3 micelles in PBS containing 0, 5, or 10 mM DTT.

In vitro release experiments with DOX-loaded micelles were carried out in PBS (pH 7.4), and micelles were subjected to redox stimulation by using the reducing substance DTT. The cumulative release (%) of DOX was also measured in PBS. For DOX-loaded polymer 3 micelles, Figure 6 shows that only 16% of the DOX was released in the buffer solution within 48 h. In contrast, more than 60% encapsulated DOX molecules were released from DOX-loaded polymer 3 micelles within 48 h in the presence of 5 and 10 mM DTT. It can be demonstrated that the disulfide linkages in these micelles could be broken under a high concentration of reducing agent, which destabilized the structure of micelles and accelerated the release of encapsulated DOX. The remaining DOX was not released, it may be that some DOX was adsorbed on the surface of the hydrophobic segment or still encapsulated due to the interaction between the hydrophobic molecules. Therefore, DOX-loaded polymer 3 micelles can meet the requirements for rapid and efficient release of the drug in a particular environment.

### 3.4. Cytotoxicity Evaluation and Inhibition of Cancer Cell

**Figure 7:**
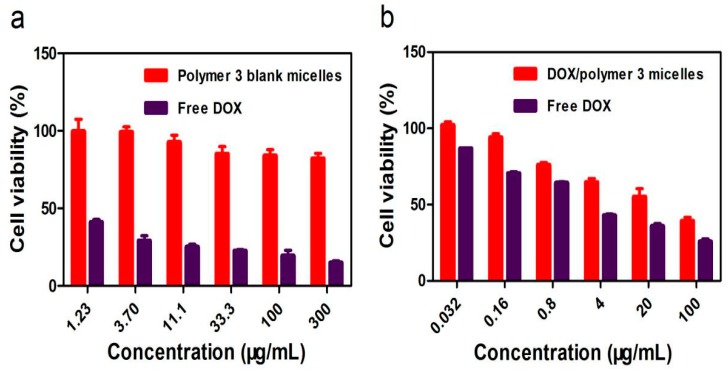
Cytotoxicity of free DOX and polymer 3 micelles in NIH-3T3 cells (**a**). Cytotoxicity of free DOX and DOX-loaded polymer 3 micelles in MCF-7 cells (**b**).

The biocompatibility of the polymeric micelles in NIH-3T3 cells was determined using CCK-8 assays. From Figure 7a, free DOX showed a higher cytotoxic effect with NIH-3T3 cells after 48 h incubation (survival rate: 20%). Cell viabilities were more than 85% for polymer 3 at the highest polymer concentration (300 µg/mL) after 48 h incubation, and this can be attributed to the excellent biocompatibility of the prepared biodegradable polymer.

As can be seen from Figure 7b, the DOX/polymer 3 micelles showed good growth inhibition efficiency in MCF-7 cells, of which 40% could survive in the polymer at a concentration of 100 µg/mL. It was possible that drug-loaded polymer 3 micelles were under high levels of GSH in MCF-7 cells, as the polymer micelle structures were destroyed within 48 h and the encapsulated DOX was released. Compared with free DOX, DOX-loaded polymer 3 micelles showed lower cytotoxicity with MCF-7 cells under the same conditions. This was due to the delayed process of the redox-triggered release of DOX from the DOX-loaded micelles in cancer cells. The results showed that the designed amphiphilic polymer micelles had excellent biocompatibility and low cytotoxicity, which may be beneficial for their use as degradable biomaterials in the delivery of anticancer drug applications.

## 4. Conclusions

In summary, three polymers were successfully synthesized, and polymer 3 micelles showed the best performance regarding drug loading. This backbone structure with numerous ester bonds was easily decomposed in bioesterase. DOX was loaded and encapsulated into the core of micelles during the self-assembly process of polymer 3 in an aqueous medium, and the DLE and DEE of DOX-loaded polymer 3 micelles were 10.39% and 58.1%, respectively. DLS experiments showed that polymer 3 micelles displayed small particle size (about 176 nm) and low CMC (5.0 mg/L). It was observed using TEM that DOX-loaded polymer 3 micelles can preserve the core-shell structure to enhance their stability, and 60% of DOX can be released effectively and rapidly in a reducing environment. In the cytotoxicity assays, polymer 3 micelles were less toxic for NH-3T3 cells (survival rate: 85%), and these drug-loaded polymer 3 micelles could effectively inhibit the proliferation of MCF-7 cells (survival rate: 40%). Therefore, these redox-responsive polymer micelles provide a new strategy for cancer chemotherapy.

## Figures and Tables

**Table 1:** Molecular weight and particle size and polydispersity (PDI) of the polymers.

Sample	M_n_,_NMR_^1^	M_n_,_GPC_^2^	M_w_/M_n_
AD-(LA_60_-co-GA_20_)_4_	11062	9789	1.39
Polymer 1	19586	16367	1.44
PT-(LA_75_-co-GA_25_)_4_	14254	11277	1.23
Polymer 2	20685	18504	1.21
AD-(LA_75_-co-GA_25_)_4_	14138	12600	1.48
Polymer 3	21652	19400	1.38

^1^ Calculated with equation M_n_,_NMR_ = (72 × x + 58 × y + 26 × m) + 912, where x, y, and m are the degree of polymerization of LA, GA, and mPEG, respectively, calculated from ^1^H NMR spectra. ^2^ Determined by GPC with tetrahydrofuran as the eluent (1.0 mL/min).

**Table 2:** Characteristics of DOX-loaded polymer micelles.

Micelle	DOX (mg)/polymer (mg)	DLE (%)	DEE (%)	Size (nm) ^1^	PDI
DOX/polymer 1	10/50	6.66	39.7	132	0.123
DOX/Polymer 2	10/50	8.94	49.1	174	0.118
DOX/Polymer 3	10/50	10.39	58.1	196	0.138

**^1^** Measured by dynamic light scattering (DLS).
